# Effect of the Intaglio Surface Treatment and Thickness of Different Types of Yttria-Stabilized Tetragonal Zirconia Polycrystalline Materials on the Flexural Strength: In-Vitro Study

**DOI:** 10.3390/ma17215256

**Published:** 2024-10-29

**Authors:** Razan S. Almirabi, Khaled M. Alzahrani

**Affiliations:** 1 Department of Prosthodontics, Riyadh Elm University, Riyadh 12734, Saudi Arabia; 2 Jeddah Second Health Cluster, Jeddah 23816, Saudi Arabia; 3 Department of Prosthetic Dental Sciences, College of Dentistry, Prince Sattam Bin Abdulaziz University, Alkharj 11942, Saudi Arabia; dr_kmq@hotmail.com

**Keywords:** Y-TZP materials, flexural strength, zirconia thickness, surface treatment, Zircos-E etching, Er:YAG laser, air abrasion

## Abstract

Background: Surface treatment of the intaglio surface of zirconia is important for bonding. However, it could affect the strength of the materials. The purpose of this study is to compare the effect of laser, etching, and air abrasion surface treatment methods to a control group on the flexural strength of three zirconia materials with two different thicknesses. (1) Methods: A total of 120 disks were divided into three groups according to the type of zirconia and the ceramic thickness. Then, according to the surface treatment method, the groups were divided into four subdivisions. The change in the microstructure of the ceramic material was investigated through Scanning Electron Microscope (EVO LS10, Carl Zeiss SMT Ltd. Oberkochen, Germany). Phase identification was performed using an X-ray diffraction device (XRD; Ultimate IV X-ray Diffractometer, Rigaku Inc., Tokyo, Japan). The flexural strength was assessed with a biaxial flexural strength test in a universal testing machine. Data were analyzed using SPSS Software (SPSS version 26.0.Armonk, NY: IBM Corp). A three-way ANOVA and a post hoc Dunnett T3 test were employed to evaluate the effect of the yttria concentration, thickness, and surface treatment on the flexural strength of zirconia (α = 0.05). (2) Results: At 0.8 mm thickness, air abrasion significantly increased the flexural strength of 3Y-TZP (1130.6 ± 171.3 MPa) and 4Y-TZP (872 ± 108.6 MPa). However, air abrasion significantly decreased the flexural strength of 5Y-TZP materials (373 ± 46.8 MPa). Laser irradiation significantly decreased the flexural strength of 5Y-TZP (347 ± 50.3 MPa), while etching significantly decreased the flexural strength of both 3Y-TZP (530 ± 48.8) and 4Y-TZP (457.1 ± 57.3). When the thickness increased to 1 mm, air abrasion continued to significantly decrease the flexural strength of 5Y-TZP materials. (3) Conclusions: There was a negative effect of surface treatment on the flexural strength at 0.8 mm thickness rather than at 1 mm thickness. Air abrasion enhances the flexural strength of 3Y-TZP and 4Y-TZP materials but significantly reduces the flexural strength of 5Y-TZP materials. Zircos-E etching and Er:YAG surface treatment methods did not significantly reduce the flexural strength of 5Y-TZP materials at 1 mm thickness and can be recommended as an alternative surface treatment for 5Y-TZP materials.

## 1. Introduction

Zirconia (zirconium dioxide) is a polycrystalline ceramic with high strength and exceptional toughness. Depending on the temperature, it forms in three crystallographic phases: the monoclinic phase occurs from room temperature to 1170 °C, the tetragonal phase occurs at temperatures from 1170 to 2370 °C, and the cubic phase occurs at temperatures above 2370 °C [1].

Among monolithic restorations, yttria-stabilized tetragonal zirconia polycrystal (Y-TZP) is characterized by excellent performance because of its high strength (>1000 MPa) and high fracture toughness (4–5 MPa·m1/2) [2]. The first monolithic zirconia used as a restorative material was composed of approximately 3 mol% yttria (3Y). This material became preferred for posterior crowns because it had high fracture strength but lacked the translucency needed for anterior restorations [3]. Some microstructural modifications can be performed to obtain a higher zirconia translucency, such as decreasing the alumina content, increasing the density, decreasing the grain size, adding cubic zirconia, and decreasing the number of impurities and structural defects [4]. To increase the content of cubic grains, the yttria content needs to be increased, which is why manufacturers have increased the yttria content of their products to approximately 5 mol% (5Y). This stabilizes the materials at 50% cubic and 50% tetragonal phases. The optical properties of these materials are more like those of lithium disilicate glass-ceramics, but their mechanical properties are much poorer than those of 3Y materials. More recently, 4Y materials have been developed by reducing the cubic phase to approximately 30% [3]. Higher amounts of yttria correspond to greater numbers of crystals fully stabilized in the cubic phase, which result in less tetragonal-to-monoclinic phase transformation in the case of mechanical stresses or cracks. This may have a negative impact on the clinical survival time of 5Y zirconia materials compared to 3Y zirconia; however, evidence is still lacking [5].

Because of its superior fracture strength, monolithic zirconia can be fabricated with minimum thickness. According to previous studies, monolithic zirconia crowns with a thickness of 1.0 mm may be similar in strength to traditional metal–ceramic crowns. However, these studies did not consider the effect of surface modifications such as grinding and airborne-particle abrasion [6]. Sorrentino et al. [7] did not find the occlusal thickness of a monolithic zirconia crown to have a significant effect on the fracture resistance of the restoration or the mode of failure. However, Abdulmajeed et al. [8] reported that when the yttria percentage is increased in the monolithic zirconia materials, increasing the thickness to at least 1.2 mm is required to improve the fracture load.

Prott et al. [9] evaluated the effects of thickness reduction and fatigue on the failure load of 3Y monolithic zirconia crowns. They studied ceramic thicknesses of 0.5, 0.8, 1.0, and 1.5 mm, in addition to a 2 mm thickness used as the control. All crowns were tested under mouth-motion fatigue using a chewing simulator with simultaneous thermocycling. They found that all tested crowns withstood failure loads above physiological chewing forces (50–250 N physiological, 500–900 N parafunctional). They also observed a significant decrease in characteristic strength for the 0.5 and 0.8 mm groups after fatigue. They concluded that a crown thickness of 1 mm or more, supported by dentin, would be less prone to fracture. However, their study did not consider surface treatment before cementation, which may affect the strength of reduced-thickness crowns. Ozer et al. [6] considered thicknesses of 0.8 mm and 1.3 mm in evaluating the flexural strength of thin restorations. They found that increasing the thickness from 0.8 mm to 1.3 mm significantly improved flexural strength, regardless of the surface treatment. They also found that monolithic zirconia thicknesses of 0.8 and 1.3 mm could withstand masticatory forces and that airborne-particle abrasion (APA) increased the flexural strength of monolithic zirconia. They suggested that APA of 0.8 mm zirconia could be beneficial because APA on conventional zirconia causes a transformation from the tetragonal to the monoclinic phase, which presumably increases the longevity of zirconia ceramic restorations. However, it has been reported that a high monoclinic phase transformation eventually reduces mechanical stability and that zirconia may lose its ability to stop crack propagation, either partially or completely, as excessive t–m phase transformation causes zirconia grains to detach and the material’s surface to delaminate, leading to catastrophic failure.

Surface treatment of the intaglio surface of zirconia crowns is used to improve the bonding of zirconia to the resin cement, which works by increasing the wettability and micromechanical interlocking [10]. Airborne-particle abrasion using alumina at high pressure is the most commonly used surface treatment as it is known to produce the roughest surface needed to obtain good adhesion [11]. However, it has been suggested that APA weakens brittle materials by creating small defects and microcracks. This point is controversial, with conflicting data having been published [3]. On the contrary, it has been reported that sandblasting surface treatment improves the flexural strength of the conventional 3Y-TZP due to the compressive layer generated by the phase transformation from tetragonal to monoclinic [12]. However, this structural alteration could render the material more susceptible to low-temperature degradation and deterioration of the compressive layer along the crack, eventually leading to the failure of the restoration [13]. Researchers have reported that an excessive increase in the monoclinic content (>12–‍25%) is responsible for the degradation of zirconia material [14,15]. Inokoshi et al. [16] found that Al_2_O_3_ sandblasting tends to increase the biaxial flexural strength of the new generations of zirconia (4Y-PSZ and 5Y-PSZ zirconia), except for the 6Y highly translucent zirconia. This was directly related to the zirconia phase composition and microstructure. Studies have also shown that 4Y zirconia is slightly strengthened by airborne-particle abrasion with alumina, while 5Y zirconia is weakened by this surface treatment [3,5]. This negative effect of airborne-particle abrasion on the 5Y-TZP strength is due to the lack of transformation toughening in this type of zirconia [17].

Different surface conditioning methods have been introduced to enhance zirconia bonding; one of these methods is laser irradiation. The use of lasers as a surface conditioning method has been researched to promote alteration in the superficial layer by laser energy discharge, which causes microexplosions, vaporization, and the melting of the superficial layer of the ceramic [18]. However, the efficiency of laser in improving bond strength remains a subject of debate [10]. Hatami et al. [19] evaluated the effect of three laser surface treatments (CO_2_, Nd:YAG, and Er:YAG) on the shear bond strength of zirconia ceramic to resin cement; they observed an increased bond strength for the ceramic specimens treated with the Er:YAG laser and they concluded that its effect on the zirconia surface was equivalent to that of sandblasting. Da Silva et al. [20] studied the effect of Er:YAG laser irradiation surface treatments with different pulse widths applied to 3Y-TZP material at a partially sintered stage. The results showed that laser irradiation decreased the mean flexural strength of zirconia at all pulse widths. Cavalcanti et al. [21] reported that irradiating zirconia surface by Er:YAG laser at an energy intensity of 400 or 600 mJ produces marked morphology alterations along with deep cracks in the ceramic surface. They observed that only 200 mJ laser energy could produce superficial surface roughness without deteriorating the ceramic surface.

Another method for increasing the surface roughness to enhance the bonding is the acid etching of zirconia. Hydrofluoric acid etching is usually performed on glass-ceramics. It acts by removing the silica-containing glassy matrix to expose the crystalline phase. However, zirconia is a polycrystalline structure that is free of silica content, which means that it is resistant to etching by hydrofluoric acid [22]. Recently, a chemical solution known as Zircos-E etching has been introduced for the etching of zirconia. It is a mixture of hydrofluoric acid (HF), hydrochloric acid (HCl), sulfuric acid (H_2_SO_4_), nitric acid (HNO_3_), and phosphoric acid (H_3_PO_4_) [23,24]. It is believed to create surface microporosity in the ceramic surface to improve the bonding strength of zirconia [25]. Cho et al. [26] studied the shear bond strength of three different methods of surface treatment. The result confirmed that the etching methods achieve higher bond strength than air abrasion and tribochemical silica coating. Nasr et al. [27] investigated the shear bond strength of ultra-translucent zirconia after two surface treatment modalities: Zircos E-etching and sandblasting. Their results showed that the etched specimens had significantly higher shear bond strengths than the control group and slightly lower bond strengths than the sandblasted specimens.

The flexural strength of ceramic materials is used to evaluate the clinical performance of dental restorations, as it reflects the material’s resistance to bending stress, which is a commonly occurring type of stress in prosthetic dentistry [28]. There are two techniques for measuring ceramic flexural strength: uniaxial and biaxial flexion [29].

The most commonly used tests for measuring the strength of dental ceramic materials are the biaxial flexural strength tests because they offer some advantages over uniaxial flexural strength tests as they create multiaxial stress states and eliminate edge failures [30]. In addition, the sample fabrication for uniaxial flexural testing methods could generate flaws on the edges that lead to variations in the strength values of the test when the load is applied [31]. Biaxial strength tests of dental ceramics are generally considered more reliable in stimulating stresses applied to the dental materials in the oral environment as they are exposed to multiaxial forces [32]. The piston-on-three-ball test has been selected by the International Organization for Standardization (ISO 6872) to assess the biaxial flexural strength of dental ceramics [33]. This selection is justified as this test does not require the two sides of the disk to be completely planar; the three-ball supports can accommodate a slightly warped specimen [34].

To the best of the author’s knowledge, no previous study has compared the effects of airborne-particle abrasion, laser, and etching surface treatment methods on the flexural strength of 3Y-TZP, 4Y-TZP, and 5Y-TZP materials. The null hypothesis to be tested is that Er:YAG laser irradiation, Zircos-E etching, and airborne-particle abrasion surface treatment methods have no significantly different effects on the flexural strengths of the three zirconia materials with different yttria concentrations at the two thicknesses.

## 2. Materials and Methods

This in vitro study included 3 different types of zirconia materials according to the yttria percentage, 3Y-TZP, 4Y-TZP, and 5Y-TZP, which are presented in Table 1.

**Figure 1:**
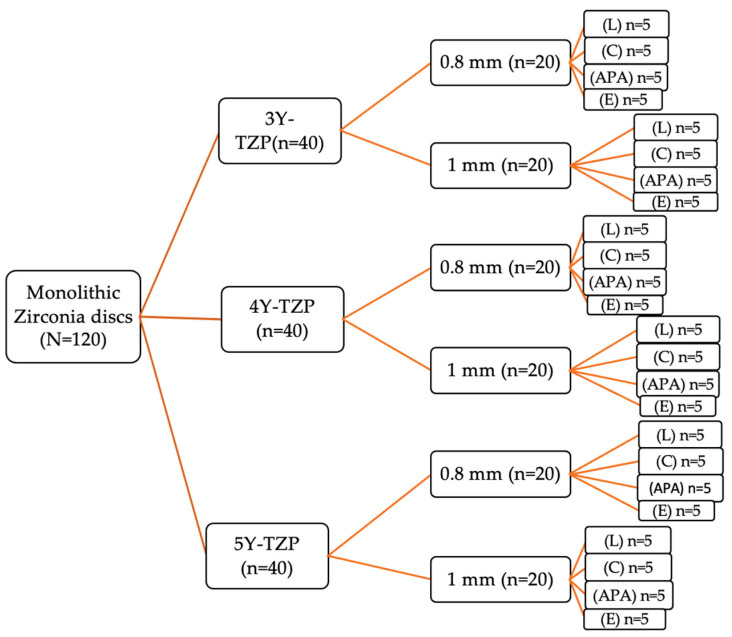
Schematic representation of the grouping of different zirconia materials with different thicknesses to be subjected to different surface treatments.

Each group was further subdivided into two subgroups (*n* = 20) according to the ceramic thickness: 0.8 mm and 1 mm. They were further divided into four subdivisions (*n* = 5), according to the surface treatment before cementation: air abrasion with 50 μ Al_2_O_3_ particles (APA), etching with Zircos–E etching solution (Zircos-E; Bioden, Seoul, Republic of Korea) (E), Er:YAG laser surface treatment (Fidelis Plus III, Fotona, Ljubljana, Slovenia) (L), and no surface treatment, which served as control (C). A schematic representation of the grouping is shown in Figure 1. Another 24 disks of 1 mm thickness were divided into two groups. Each group has 4 specimens from each zirconia type. One group (*n* = 12) was used for the surface microstructure analysis using SEM. The other group (*n* = 12) was used for the phase characterization using XRD.

Monolithic zirconia disks were milled with a 5-axis milling machine (Roland DWX-50 5-Axis Dental Mill, Roland, DGA, Corporation, Irvine, CA, USA) from pre-sintered blanks of each material. Then, specimens were sintered at 1450 °C according to the manufacturer’s instructions. Then, they were dry polished by hand with 1200-grit silicon carbide paper. A digital caliper was used to measure the specimens’ dimensions.

Before surface treatment, the samples were ultrasonically cleaned in distilled water for 10 min and then air-dried. The control group (C) received no surface treatment. The APA group (APA) was subjected to air abrasion with 50 μm aluminum oxide particles using sandblaster, the nozzle was placed at 90° incidence angle from the center of the disk, and the duration was 20 s under a pressure of 2 bar from a distance of 10 mm [35]. In the laser group (L), the surfaces of zirconia disks were covered with graphite powder and irradiated with Er:YAG laser (Fidelis Plus III, Fotona, Ljubljana, Slovenia) with a wavelength of 2.94 μm wavelength. Laser parameters were set as follows: 50 μs pulse duration (SSP), 2 W output power, 10 Hz pulse repetition, and 200 mJ energy. The laser was delivered through a tipless (non-contact) 90°-angled handpiece that was adjusted manually at approximately 1 mm distance from the sample, perpendicular to the disk surface, and the ceramic surface irradiation was carried out for 10 s using horizontal scanning mode. Fine air and water cooling were used during the irradiation of the samples [19]. For the etching group (E), the disks were submerged in the Zircos-E etching solution (Zircos-E, Bioden. Co., Seoul, Republic of Korea) for two hours. They were then rinsed under cold tap water for three minutes, followed by steam cleaning and an annealing process to relieve the residual stresses and remove any residual substances [24,25,27]. After surface preparation, the disks were cleaned in distilled water in an ultrasonic bath and then air-dried.

### 2.1. Biaxial Flexural Strength Test (BFS)

The biaxial flexural strengths were evaluated using a universal testing machine (Instron 5965, Instron Corporation, Norwood, MA, USA). The specimens were placed on a fixture with three equidistance stainless steel spherical balls measuring 3.2 mm in diameter, distributed on the periphery of a 10 mm diameter circle with an angle of 120° between the steel balls. A cylindrical head piston with a diameter of 1.2 mm applied a load at a rate of 1 mm/min to the opposite side of the treated surface to place the treated surface under flexural tension (ISO6872:2015) [33]. The load in newtons (N) required to fracture the specimens was automatically recorded, and the BFS was calculated using the following equations:

S = −0.2387P (X − Y)/d^2^

X = (1 + v)In(r_2_/r_3_)^2^ + ([1 − v]/2) (r_2_/r_3_)^2^

Y = (1 + v) (1 + In[r_1_/r_3_]^2^) + (1 − v)(r_1_/r_3_)^2^

where S is the biaxial flexural strength (MPa), P is the fracture load (N), d is the thickness of the disk (mm), v is Poisson’s ratio (0.25), r_1_ is the radius of the support circle (5 mm), r_2_ is the radius of the loaded area (0.6 mm), and r_3_ is the radius of the specimen (6 mm).

### 2.2. Scanning Electron Microscope (SEM)

Changes in the microstructure of the ceramic material were investigated through SEM (EVO LS10, Carl Zeiss SMT Ltd. Oberkochen, Germany). The samples were rinsed with 96% ethanol and air-dried. Then, they adhered on the sample stub using double-sided adhesive conductive carbon tape (Agar Scientific, Stansted, UK) facing the treated surface upside and then sputter-coated with gold using a sputter coater (Q150RS, Quorum Technologies, Laughton, East Sussex, UK). The images of the gold-coated samples were taken at different magnifications (X1000, 2000, and 4000) using 15 kV EHT voltage.

### 2.3. X-Ray Diffraction (XRD)

Phase identification was performed using an X-ray diffraction device (XRD; Ultimate IV X-ray Diffractometer, Rigaku Inc., Tokyo, Japan) to evaluate the relative percentage of the monoclinic phase on the treated specimens. The tube anode was Cu with Ka = 0.1540562 nm monochromatized with a graphite crystal. The pattern was operated at 50 kV of tube voltage, over 5−80° degrees, 2θ range, and 140 mA of tube current in step scan mode, with 0.02° step size and counting time of 1 s per step.

### 2.4. Statistical Analysis

Collected data were evaluated using Statistical Package for the Social Sciences (SPSS version 26.0.Armonk, NY: IBM Corp). The normal distribution of data was confirmed using Shapiro–Wilk test (*p*-Value > 0.05). Descriptive statistics were performed for all the groups, and three-way analysis of variance (ANOVA) was employed to evaluate the effect of yttria mol% concentration, material thickness, and surface treatment on the flexural strength of zirconia, and post hoc Dunnett T3 multiple comparison test was used for pairwise comparisons. A significance level of *p*-value ≤ 0.05 was considered for all the tests.

## 3. Results

### 3.1. Biaxial Flexural Strength (BFS)

**Figure 2:**
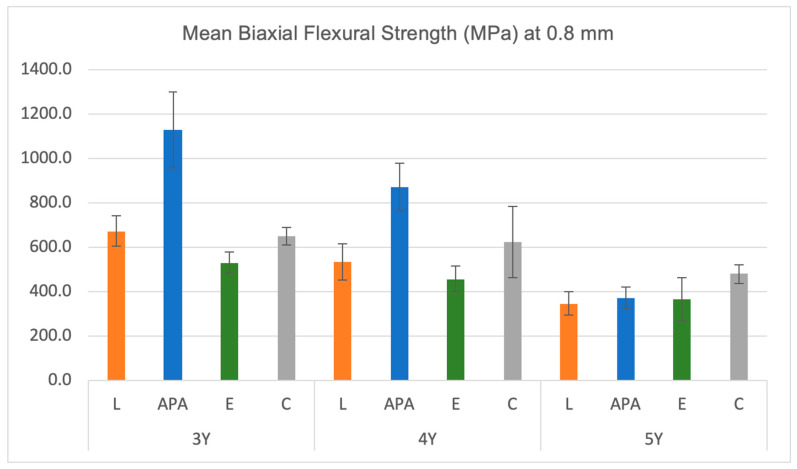
Descriptive statistics of BFS values (MPa) for the three materials after different surface treatments at 0.8 mm thickness. Error bars indicate the standard deviations. L = laser, APA = air abrasion, E = etching, C = control.

**Figure 3:**
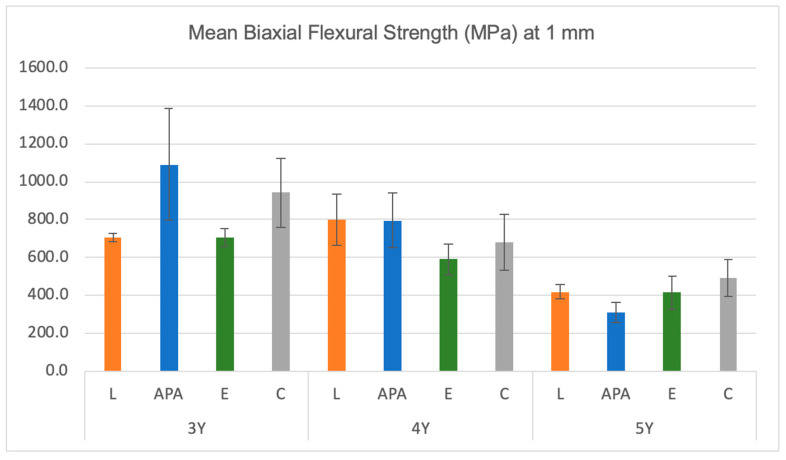
Descriptive statistics of BFS values (MPa) for the three materials after different surface treatments at 1 mm thickness. Error bars indicate the standard deviations. L = laser, APA = air abrasion, E = etching, C = control.

The descriptive statistics of the mean biaxial flexural strength values (in megapascal) for the three materials after various surface treatment techniques at 0.8 mm thickness are presented in Table 2 and Figure 2. Similarly, the descriptive statistics of the mean biaxial flexural strength values for the three materials after various surface treatment techniques at 1 mm thickness are shown in Table 3 and Figure 3.

The ANOVA test results presented in Table 4 indicate that at a thickness of 0.8 mm, there was a significant difference in the BFS values between the materials after different surface treatments (*p* < 0.05). At 1 mm thickness, a significant difference was found between 3Y and 5Y materials (*p* < 0.05), but no significant difference was observed for the 4Y material (*p* > 0.05). At the thickness of 0.8 mm, multiple comparisons revealed that for the 3Y-TZP material, the (APA) group exhibited significantly higher BFS values than all the other groups (1130.6 ± 171.3 MPa). For the 4Y-TZP material, the (APA) group showed significantly higher BFS values (872.4 ± 108.6 MPa) than the (L) (534.3 ± 80.1 MPa) and (E) (457.1 ± 57.3 MPa) groups. In contrast, for the 5Y-TZP material, the (APA) group exhibited significantly lower BFS values (373 ± 46.8 MPa) than the (C) group (479.3 ± 43.4 MPa). Similarly, the (L) group showed significantly lower BFS values (347 ± 50.3 MPa) than the (C) group. However, there is no statistically significant difference between the (E) and (C) groups (*p* > 0.05). At 1 mm thickness, multiple comparisons indicated that for 3Y-TZP and 4Y-TZP materials, there is no statistically significant difference between all the surface treatments on the BFS values (*p* > 0.05). However, for the 5Y-TZP material, the (APA) group demonstrated significantly lower BFS values (309.1 ± 52.3 MPa) than the (C) group (490.5 ± 96.5 MPa).

### 3.2. Scanning Electron Microscope (SEM)

**Figure 4:**
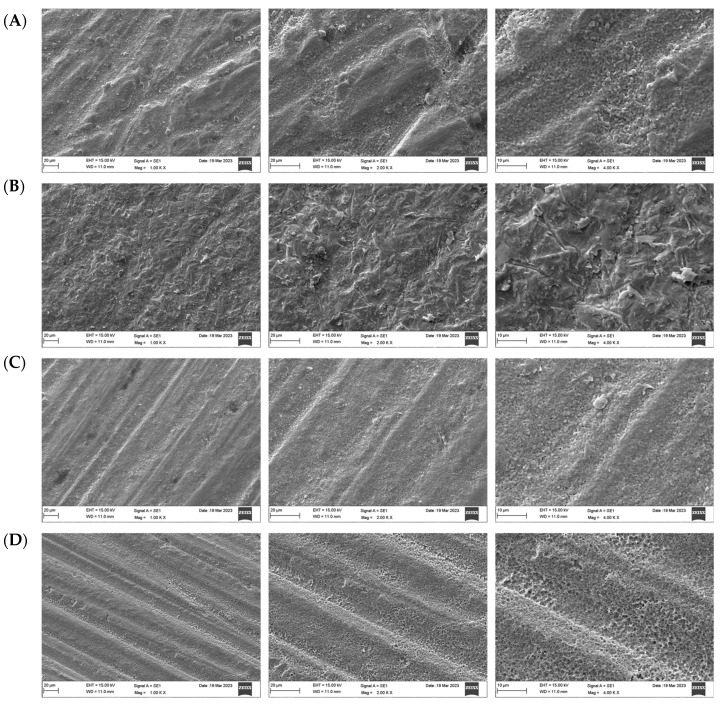
Surface topography of 3Y-TZP after different surface treatments under magnifications (×1000, 2000, 4000), respectively. (**A**) Control, (**B**) airborne-particle abrasion, (**C**) laser, (**D**) etching.

**Figure 5:**
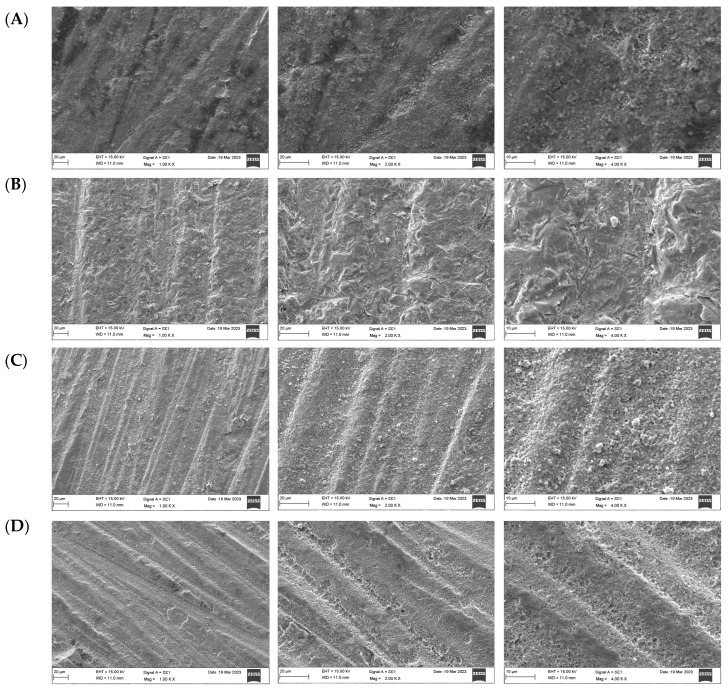
Surface topography of 4Y-TZP after different surface treatments under magnifications (×1000, 2000, 4000), respectively. (**A**) Control, (**B**) airborne-particle abrasion, (**C**) laser, (**D**) etching.

**Figure 6:**
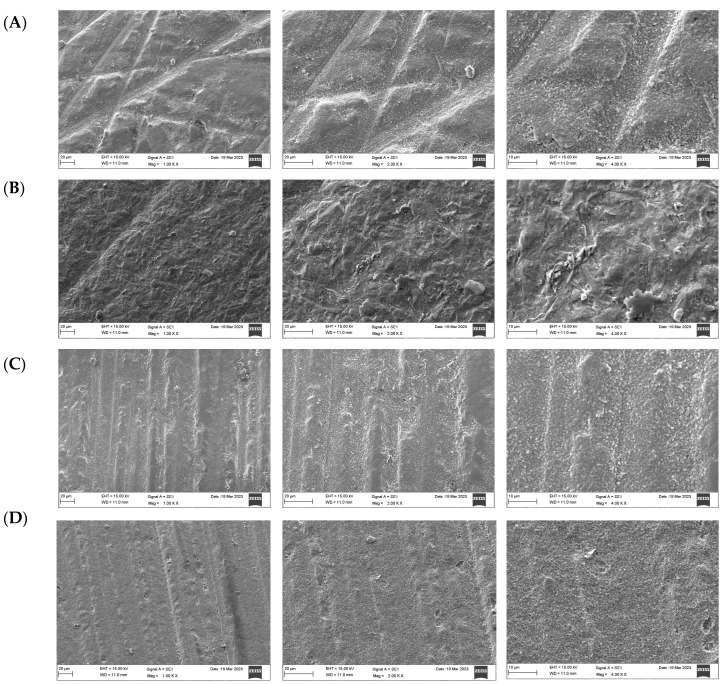
Surface topography of 5Y-TZP after different surface treatments under magnifications (×1000, 2000, 4000), respectively. (**A**) Control, (**B**) airborne-particle abrasion, (**C**) laser, (**D**) etching.

The SEM micrographs presented in Figure 4, Figure 5 and Figure 6 illustrate the differences in surface topography among the Y-TZP samples subjected to distinct surface treatment techniques. The control specimens demonstrated multiple grooves, indicating machined and polished surfaces (Figure 4A, Figure 5A and Figure 6A). The air abrasion surface treatment produces large irregular peaks and valleys and superficial scratches in the ceramic surfaces (Figure 4B, Figure 5B and Figure 6B). The laser-treated group showed surface imperfections and deep fissures in a homogeneous pattern (Figure 4C, Figure 5C and Figure 6C). In turn, the acid-etched samples showed a uniform coarse appearance and numerous tiny grooves (Figure 4D, Figure 5D and Figure 6D).

### 3.3. X-Ray Diffraction (XRD)

In this study, the crystalline phases in the samples were analyzed using the HighScore Plus software (version 5.1) with the JCPDS Cards 00–048-0224 (tetragonal lattice), 00–037-1484 (monoclinic lattice), 01–070-4436 (cubic lattice), and 00–037-1307 (rhombohedral lattice). The amounts of the monoclinic, cubic, and tetragonal phases were determined through a standard Rietveld refinement using the FullProf Suite software (https://www.ill.eu/sites/fullprof/), where only the scale factor of the crystalline phases was refined for each sample to ensure consistent results.

**Figure 7:**
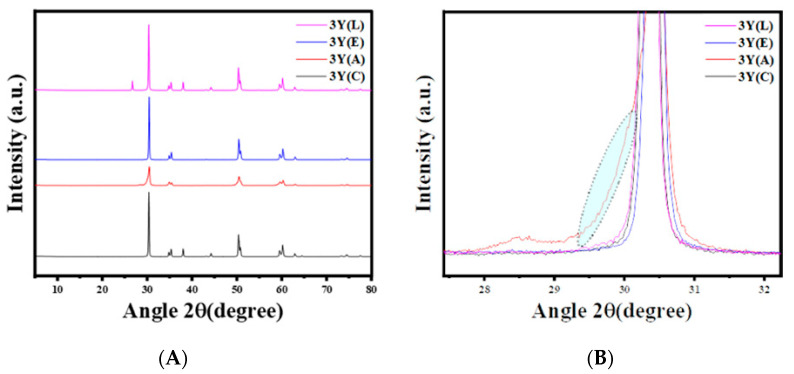
Representative XRD patterns of 3Y-TZP after different surface treatments. (**A**) 3Y-TZP groups, (**B**) monoclinic peak emergence after air abrasion.

**Figure 8:**
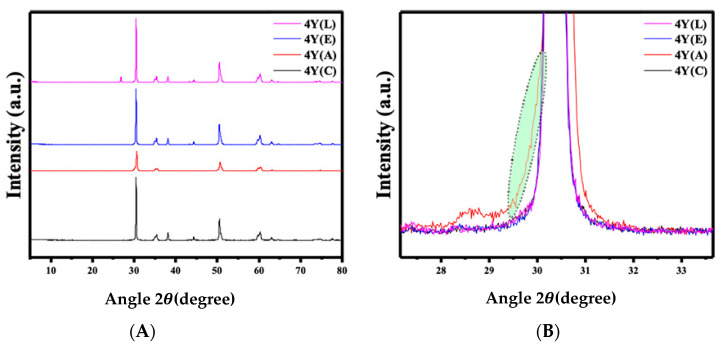
Representative XRD patterns of 4Y-TZP after different surface treatments. (**A**) 4Y-TZP groups, (**B**) appearance of a rhombohedral phase in 4Y (A) group.

**Figure 9:**
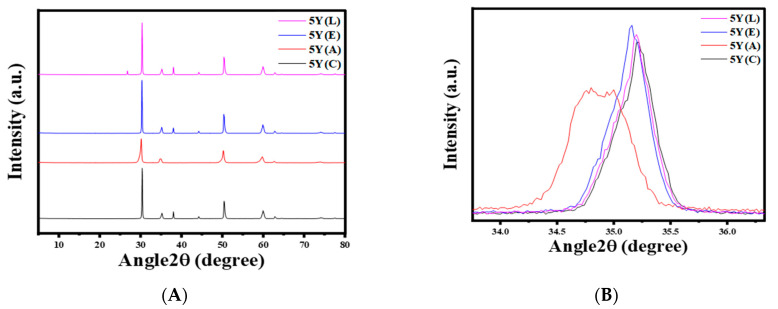
Representative XRD patterns of 5Y-TZP after different surface treatments. (**A**) 5Y-TZP groups, (**B**) broadening of the primary intensity of the tetragonal phase peak in 5Y (A).

The XRD patterns of each zirconia material are presented in Figure 7, Figure 8 and Figure 9. The 3Y materials are presented in Figure 7. The air abrasion of the specimens resulted in the emergence of a monoclinic peak at 2θ = 28.44°, which can be noticed in Figure 7B, indicating that the sandblasting process caused changes in the structure of the ceramic surface. The (APA) group shows the highest amount of the monoclinic phase with 6.91 (wt.%), while other groups have not shown any monoclinic peak. In the 4Y materials presented in Figure 8, a monoclinic peak was observed at 2θ = 30.54° following the air abrasion surface treatment. The (APA) group exhibited the highest monoclinic phase concentration of 3.26 (wt.%), while no monoclinic peak was observed in the other groups. For the 5Y materials shown in Figure 9, no monoclinic peak was detected after any of the surface treatments. However, a broadening of the primary intensity of the tetragonal phase peak was observed in the 5Y(A) group.

## 4. Discussion

This study was conducted to evaluate the effect of Er:YAG laser irradiation, Zircos-E etching, and airborne-particle abrasion surface treatment methods compared to a control group on the flexural strength of three zirconia materials with different yttria concentrations (3%, 4%, and 5%) at two different thicknesses (0.8 and 1 mm). The results proved that the tested surface treatment methods significantly affected the flexural strength of the different types of zirconia on different thicknesses; thus, the null hypothesis was rejected.

Sorrentino et al. [7] found that the occlusal thickness of zirconia crowns did not influence either the fracture resistance or the failure mode of restorations. However, in the present study, the negative effect of surface treatment on the zirconia flexural strength decreased in thicker specimens. For the 5Y-TZP materials, all the surface treatment methods showed a reduction in the flexural strength, which emphasizes that additional tooth preparation is usually needed for these materials. As reported by Abdulmajeed et al. [8], a minimum material thickness of 1.2 mm is needed for 5Y materials.

Previous studies [12,13,31,35] have shown that a sandblasting surface treatment significantly enhances the flexural strength of conventional zirconia 3Y-TZP because of the transformation toughening mechanism. Similarly, our study results show that airborne–particle abrasion increases the flexural strength of 3Y materials. The results also show that airborne–particle abrasion increases the flexural strength of 4Y materials to a lesser extent but significantly reduces the strength of 5Y materials. These findings are consistent with those of previous studies [3,5] that showed that the transformation toughening mechanism is inversely related to the yttria content. In contrast, Inokoshi et al. [16] showed that the biaxial flexural strength increased after sandblasting for 4Y-PSZ and 5Y-PSZ but decreased for 6Y-PSZ. Sulaiman et al. [13] suggested that even though the biaxial flexural strength is higher after sandblasting, the reliability of the material could be diminished by increased susceptibly to low-temperature degradation.

In the present study, Er:YAG laser did not significantly affect the flexural strength of 3Y and 4Y materials. In contrast, da Silva et al. [20] reported that it significantly reduced the flexural strength of 3Y-TZP at all studied pulse widths. However, these results might have been due to the application of the surface treatment while the zirconia was at the partially sintered stage. A systematic review concluded that Er:YAG laser irradiation is not an effective alternative for enhancing the zirconia bond strength to resin cement or veneering ceramic [18]. These conflicting results could be related to the variations in laser parameters and the application of graphite powder.

Zircos-E etching surface treatment did not significantly affect the flexural strength of the materials at 1 mm thickness. Ansari et al. [23] found that Zircos-E etching enhanced the bond strength of translucent zirconia only, which they attributed to the phasic structure of highly translucent zirconia materials. They also found that this etching solution did not affect the hardness of the material. In the present study, we found that, unlike air-particle abrasion, Zircos-E etching did not significantly reduce the flexural strength of 5Y-TZP materials.

A surface microstructure analysis using SEM revealed significant differences between the samples. As reported in a previous study [25], an air abrasion surface treatment produces the characteristic appearance of a sandblasted surface with irregular surface roughness and large irregular peaks and valleys with sharp edges, which could be due to the aluminum oxide particles hitting the ceramic surface randomly. Consistent with the findings of a previous study [15], no mechanical flaws, cracks, or defects were noticed. Laser-irradiated surfaces demonstrated high surface roughness, and these results are consistent with those obtained by Hatami et al. [19], who found that laser increased surface roughness and thereby enhanced the bond strength of zirconia surfaces to resin cement without damaging the ceramic surface. The samples etched with Zircos-E showed several tiny grooves, along with a uniformly coarse appearance; these grooves had the characteristic appearance of a chemically treated surface, suggesting the creation of micropores without aggressive roughening. Similar findings were reported by Sales et al. [25].

Regarding the phase characterization after different surface treatments, our findings indicated that the concentration of yttrium is inversely related to the intensity of the monoclinic peak, which decreased with increasing yttrium concentration. This was also observed by Kim et al. [22], who attributed the absence of the monoclinic phase in some of the 4Y and 5Y material subgroups after air abrasion to the decreased amount of the tetragonal phase and the increased amount of the rhombohedral phase. It has also been reported that the emergence of this phase after sandblasting is responsible for the weakening of translucent types of zirconia [22]. The presence of this phase in the present study indicated that there are subsurface changes in all the zirconia materials after air-particle abrasion.

Furthermore, Inokoshi et al. [16] reported that the transformability of the t-ZrO_2_ phase decreased with increasing yttria content, which was attributed to the reduced enhancement of flexural strength due to the reduction in compressive stress. Nevertheless, according to the literature, if the monoclinic content generated by sandblasting is in the range of 12–25%, the monoclinic-to-tetragonal transformation only occurs in the outer surface of the material and does not affect the long-term flexural strength [14,15].

Some limitations of this study should be kept in mind as this was an in vitro study that did not precisely reproduce clinical conditions. Additionally, flat zirconia specimens were used for standardization, whereas, in clinical situations, monolithic zirconia crowns are anatomically contoured.

Further in vitro studies and clinical trials under different environmental conditions are recommended to confirm the results of this study. In addition, further comparisons of the effects of the various surface treatment techniques on the bond strengths of different types of zirconia ceramics are suggested.

## 5. Conclusions

Within the limitations of the present study, the following conclusions can be drawn:

1. There was a negative effect of surface treatment on zirconia flexural strength at 0.8 mm thickness rather than at the thicker specimens.
2. Airborne-particle abrasion surface treatment enhances the flexural strength of 3Y and 4Y zirconia materials but significantly reduces the strength of 5Y zirconia materials.
3. Er:YAG laser and Zircos-E etching can be used as an alternative surface treatment for 5Y-TZP materials at 1 mm thickness without significantly affecting the materials’ flexural strength.

## Figures and Tables

**Table 1:** Materials used in the study.

Material	Chemical Composition [wt.%]	Shade	Size (mm)	Lot No.	Manufacturer
DD Bio ZX^2^—High Translucent (3Y-TZP-LA)	ZrO_2_ + HfO_2_ + Y_2_O_3_ ≥ 99.0 Y_2_O_3_ < 6 Al_2_O_3_ ≤ 0.15 Other oxides < 1.0	White	98.5 × 14	5032115008	Dental Direkt
DD cube ONE—High Translucent Plus (4Y-TZP)	ZrO_2_ + HfO_2_ + Y_2_O_3_ ≥ 99.0 Y_2_O_3_ < 8 Al_2_O_3_ ≤ 0.15 Other oxides < 1.0	White	98.5 × 14	7162202045	Dental Direkt
DD cubeX^2^—Super High Translucent (5Y-TZP**)**	ZrO_2_ + HfO_2_ + Y_2_O_3_ ≥ 99.0. Y_2_O_3_ ≤ 10 Al_2_O_3_ ≤ 0.01 Other oxides < 1.0.	White	98.5 × 14	8032110002	Dental Direkt

A total number of 144 disk-shaped specimens with a diameter of 12 mm were used in this study (*n* = 144). A number of 120 disks were divided into three groups according to the type of Y-TZP: 40 disks of 3Y-TZP, 40 disks of 4Y-TZP, and 40 disks of 5Y-TZP.

**Table 2:** Descriptive statistics of BFS values (MPa) for the three materials after different surface treatments at 0.8 mm thickness.

Thickness	Material	Surface Treatment	Mean	Std. Deviation	95% Confidence Interval for Mean	
					Lower Bound	Upper Bound
0.8 mm	3Y	Laser (L)	673.4	69.8	586.7	760.1
		Air Abrasion (APA)	1130.6	171.3	917.9	1343.2
		Etching (E)	530.7	48.8	470.2	591.3
		Control (C)	650.0	39.6	600.8	699.2
	4Y	Laser (L)	534.3	80.1	434.8	633.7
		Air Abrasion (APA)	872.4	108.6	737.5	1007.2
		Etching (E)	457.1	57.3	385.9	528.3
		Control (C)	622.6	160.2	423.6	821.6
	5Y	Laser (L)	347.0	50.3	284.6	409.5
		Air Abrasion (APA)	373.0	46.8	314.9	431.2
		Etching (E)	367.0	97.6	245.9	488.2
		Control (C)	479.3	43.4	425.4	533.2

**Table 3:** Descriptive statistics of BFS values (MPa) for the three materials after different surface treatments at 1 mm thickness.

Thickness	Material	Treatment	Mean	Std. Deviation	95% Confidence Interval for Mean	
					Lower Bound	Upper Bound
1 mm	3Y	Laser (L)	705.2	21.3	678.8	731.6
		Air Abrasion (APA)	1091.0	293.9	726.2	1455.9
		Etching (E)	707.2	47.1	648.8	765.7
		Control (C)	941.8	183.3	714.3	1169.4
	4Y	Laser (L)	798.4	136.5	629.0	967.9
		Air Abrasion (APA)	795.6	145.8	614.6	976.6
		Etching (E)	589.3	84.3	484.6	694.0
		Control (C)	680.4	146.5	498.4	862.3
	5Y	Laser (L)	416.9	37.6	370.2	463.6
		Air Abrasion (APA)	309.1	52.3	244.1	374.0
		Etching (E)	414.1	87.5	305.4	522.8
		Control (C)	490.5	96.5	370.7	610.4

**Table 4:** ANOVA test results of the effect of different surface treatments on the flexural strength of each material at different thicknesses.

ANOVA							
S							
Thickness	Material		Sum of Squares	df	Mean Square	F	Sig.
0.8 mm	3Y	Between Groups	1,043,619.649	3	347,873.216	36.471	0
		Within Groups	152,611.573	16	9538.223		
		Total	1,196,231.222	19			
	4Y	Between Groups	487,846.811	3	162,615.604	13.787	0
		Within Groups	188,720.365	16	11,795.023		
		Total	676,567.176	19			
	5Y	Between Groups	53,115.095	3	17,705.032	4.393	0.02
		Within Groups	64,488.449	16	4030.528		
		Total	117,603.544	19			
1 mm	3Y	Between Groups	536,827.717	3	178,942.572	5.837	0.007
		Within Groups	490,504.209	16	30,656.513		
		Total	1,027,331.926	19			
	4Y	Between Groups	152,272.779	3	50,757.593	2.966	0.063
		Within Groups	273,795.494	16	17,112.218		
		Total	426,068.272	19			
	5Y	Between Groups	83,561.176	3	27,853.725	5.273	0.01
		Within Groups	84,520.287	16	5282.518		
		Total	168,081.462	19			

## Data Availability

The original contributions presented in the study are included in the article, further inquiries can be directed to the corresponding author.
